# Molecular fungal community and its decomposition activity in sapwood and heartwood of 13 temperate European tree species

**DOI:** 10.1371/journal.pone.0212120

**Published:** 2019-02-14

**Authors:** Sabrina Leonhardt, Björn Hoppe, Elisa Stengel, Lisa Noll, Julia Moll, Claus Bässler, Andreas Dahl, Francois Buscot, Martin Hofrichter, Harald Kellner

**Affiliations:** 1 Technische Universität Dresden, International Institute Zittau, Department of Bio- and Environmental Sciences, Zittau, Germany; 2 UFZ-Helmholtz Centre for Environmental Research, Department of Soil Ecology, Halle (Saale), Germany; 3 Julius Kuehn-Institute, Institute for National and International Plant Health, Braunschweig, Germany; 4 University of Würzburg, Field Station Fabrikschleichach, Rauhenebrach, Germany; 5 University of Vienna, Department of Microbiology and Ecosystem Science, Vienna, Austria; 6 Bavarian Forest National Park, Grafenau, Germany; 7 Technische Universität Dresden, Center for Molecular and Cellular Bioengineering, CMCB Technology Platform, Deep Sequencing Group, Dresden, Germany; 8 German Centre for Integrative Biodiversity Research (iDiv), Halle-Jena-Leipzig, Leipzig, Germany; USDA Forest Service, UNITED STATES

## Abstract

Deadwood is an important structural component in forest ecosystems and plays a significant role in global carbon and nutrient cycling. Relatively little is known about the formation and decomposition of CWD by microbial communities *in situ* and about the factors controlling the associated processes. In this study, we intensively analyzed the molecular fungal community composition and species richness in relation to extracellular enzyme activity and differences in decomposing sapwood and heartwood of 13 temperate tree species (four coniferous and nine deciduous species, log diameter 30–40 cm and 4 m long) in an artificial experiment involving placing the logs on the forest soil for six years. We observed strong differences in the molecular fungal community composition and richness among the 13 tree species, and specifically between deciduous and coniferous wood, but unexpectedly no difference was found between sapwood and heartwood. Fungal species richness correlated positively with wood extractives and negatively with fungal biomass. A distinct fungal community secreting lignocellulolytic key enzymes seemed to dominate the decomposition of the logs in this specific phase. In particular, the relative sequence abundance of basidiomycetous species of the Meruliaceae (e.g. *Bjerkandera adusta*) correlated with ligninolytic manganese peroxidase activity. Moreover, this study reveals abundant white-rot causing Basidiomycota and soft-rot causing Ascomycota during this phase of wood decomposition.

## Introduction

Deadwood, or coarse woody debris (CWD), is an important structural component in forest ecosystems that influences a large number of ecosystem functions and contains diverse microhabitats for various organisms [1–3]. Although CWD plays an important role in the global carbon and nutrient cycle [4–6] due to its potential to store large amounts of carbon [1, 7], relatively little is known about its decomposition *in situ* and about the factors controlling the associated processes [7]. The rate of decomposition of wood is typically slower than that of leaf or litter materials [8], and coniferous CWD decomposes more slowly than deciduous CWD, as well as heartwood decomposing more slowly than sapwood [9–11].

The microbial decomposition of CWD is dominated by filamentous Dikarya fungi, especially Agaricomycotina and xylariaceous Ascomycota [5,12–16]. These organisms have the ability to break down the recalcitrant lignocellulose complex by an effective toolbox of extracellular oxidoreductases and hydrolases that are encoded in high numbers in their genomes [10,11,14,17,18]. Wood-decaying fungi are usually divided into three major ecological categories: white-rot or white-rot like fungi (WRF, also subtypes, simultaneous and selective white rot) [19], brown-rot fungi (BRF) and soft-rot fungi (SRF; type I and II), depending on modification of the lignocellulose complex and the technique used for decomposition, i.e. *via* multiple different secreted enzymes (e.g. WRF, SRF) or by the combined action of only few enzymes but strong radical-based mechanisms (e.g. BRF) [3,6,20–23].

Fungal communities change over the course of deadwood decomposition; for example, in boreal forests wood-rotting species dominate in the initial and mid phase of decomposition in *Picea abies* logs and decline in late phases, often replaced by ectomycorrhizal species [2,15,24,25]. However, this succession can vary depending on forest biome [16], tree species (e.g. *Fagus sylvatica*) [15,16] and among different CWD components like bark, sapwood or heartwood. Kubartová and coauthors [5] found significant differences in fungal richness between sapwood and heartwood in natural and artificial experiments, and for the fungal community composition in an artificial decomposition experiment of 14-year-old *Picea abies* logs. Van der Wal and coauthors [26] reported significant differences in the fungal community composition between sapwood and heartwood of *Larix kaempferi* and *Quercus rubra* after one year of decomposition but not in the following year. Aside from molecular studies, traditional sporocarp surveys have revealed that species richness of wood-decomposing fungi tends to increase with the amount of substrate [2,5,27,28] and with progressive wood decomposition [29]. Enhanced competition for space, resources or nutrients in species-rich communities may have a negative impact on the rate of decomposition, since it may limit fungal growth [5,30]. On the other hand, there are numerous studies providing evidence to support both antagonistic and synergistic relationships during fungal wood colonization [30]. These inconsistent findings emphasize the need for further studies disentangling fungal communities in more detail, e.g. by considering new tree species or by discriminating between sapwood and heartwood. This is becoming increasingly feasible as high through-put sequencing of wood-inhabiting microbial communities becomes more widely accessible and available.

The present study follows on from previous investigations using the same experimental platform—the BELongDead (biodiversity exploratories long-term deadwood) experiment, which is examining the decomposition process in logs from 13 deciduous and coniferous temperate tree species with one starting point for decay [31–35]. In this context, we investigated the in-depth relationships between fungal biomass, enzyme activities and CWD properties in sapwood and heartwood of these decomposing logs exposed for six years on the ground [10]. Fungal biomass and lignocellulolytic enzyme activity were significantly higher in sapwood than in heartwood and higher in deciduous than in coniferous species, however, with a rather weak correlation to each other. Thus, the questions remain, how the fungal community reflects the observed enzyme patterns and how this may correspond to the resulting decomposition process observed on the basis of the changes in the wood physicochemical parameters.

In the study presented here, tracking the decomposition of 13 tree species, we predicted strong differences between the 13 tree species in general, especially between coniferous and deciduous tree species (hypothesis H1). Moreover, we predicted significant differences in the fungal community composition between sapwood and heartwood, in addition to the previously observed varying amounts of fungal biomass and different enzymatic activities (H2) [10]. Furthermore, as a working hypothesis, we predicted that we would find higher species richness in association with the observed higher fungal biomass after six years of decomposition (H3). To the best of our knowledge, observations that could address these basic questions do not exist for *in situ* wood decomposition experiments (i.e. molecular field studies), even if both variables, molecular derived species richness data and ergosterol content, have been measured [15]. We aimed to gain novel insights into the fungal community composition and the respective dominant eco-physiologies. Not least, we predicted that we would see a positive relationship between the relative abundance of white-rot fungi and the activities of lignocellulolytic enzymes, which mediate the crucial process of lignin decomposition and, in parallel or subsequently, the breakdown of cellulose and hemicelluloses in wood (H4).

## Material and methods

### Field site and sample collection

Fieldwork permits were issued by the responsible state environmental offices of Thüringen (according to § 72 BbgNatSchG). The study was conducted in forest plots of the German Biodiversity Exploratories at the Hainich National Park in Central Germany (N 51.08, E 10.43) [36]. In winter 2008/2009 the BELongDead experiment was established to research deadwood decomposition [11]. Freshly cut logs of 13 temperate tree species (*Acer* sp., *Betula* sp., *Carpinus betulus*, *Fagus sylvatica*, *Fraxinus excelsior*, *Larix decidua*, *Picea abies*, *Pinus sylvestris*, *Populus* sp., *Prunus avium*, *Pseudotsuga menziesii*, *Quercus* sp. and *Tilia* sp., hereafter only genus names are used; diameter 30–40 cm and 4 m long), each with three replicates, were placed in representative research plots to allow their decomposition to be monitored during the coming decades [11]. To analyze spatial variation between sapwood and heartwood-inhabiting communities intensively, we focused on three subsets of the entire BELongDead experiment, each in a beech forest plot of the Hainich National Park (known as HEW7-9). In June 2014, sampling of 41 deadwood logs (3 x 13 logs, but on two sites, two incorrectly placed logs of *Acer* sp. and *Larix decidua* were additionally sampled) was carried out as described in Noll et al. [10]. Distinguishable sapwood and heartwood samples were collected in the form of chips by driving an auger horizontally into the log center (15–20 cm deep, 1 m distance from log end) of the selected logs. After bark removal, sapwood shavings were collected from a first drilling with a 2 cm auger (first 5 cm depth) followed by a second drilling in the same hole to collect heartwood shavings. Each log was sampled once. To avoid contamination, the auger was flamed between each deadwood sampling and samples were immediately frozen whilst awaiting analysis. As a pragmatic definition, the separation into heartwood (“older nonliving central wood”) and sapwood (“physiologically active outer portion of wood”) is defined in this study for all tree species, even if only *Fraxinus excelsior*, *Prunus avium*, *Quercus* sp., *Larix decidua*, *Pinus sylvestris* and *Pseudotsuga menziesii* are known to contain a distinct heartwood fraction.

### Enzymatic activity and fungal biomass

All CWD samples were ground and extracted as described previously in Noll et al. [10]; ligninolytic enzyme activities (laccase, peroxidase) as well as activities of other important hydrolases (enzymes involved in C, N, P-cycling) and analysis of fungal biomass were described in the same research article. For this study, we measured activities of five additional hydrolytic enzymes using water-extracted samples and a high-performance liquid chromatography (HPLC) method (Agilent Series 1200, Agilent Technologies Deutschland GmbH, Böblingen, Germany) with a modified protocol adapted from Freeman [37] and Stemmer [38]. For this purpose, five fluorogenic 4-methylumbelliferone (4-MU) derivatives, i.e. 4-MU-*β*-*D*-glucuronide, 4-MU-*α*-*D*-mannopyranoside, 4-MU-*α*-*L*-arabinopyranoside, 4-MU-phosphate and 4-MU-sulfate (Sigma-Aldrich, Steinheim, Germany) were used as assay substrates for *β*-*D*-glucuronidase (EC 3.2.1.31), *α*-*D*-mannosidase (EC 3.2.1.24), *α*-*L*-arabinosidase (EC 3.2.1.55), acid phosphatase (EC 3.1.3.2) and sulfatase (EC 3.1.6.1), respectively. A HyperClone column (ODS-C18, 5 μm, 120 Ǻ, 250 × 4 mm, Phenomenex, Aschaffenburg, Germany) was used for substrate and product separation. The analytes were eluted at 0.8 ml min^-1^ and 40°C with potassium phosphate buffer (15 mM, pH 6; A) and methanol (B). The following gradient was used: 13% B held for 6 min, increase to 20% in 2.5 min, increase to 55% in 6.5 min, increase to 75% in 1 min, and then returning to the primary conditions. Separated substrates as well as the 4-MU products were quantified at 315 nm. In all cases, the mean values of triplicate determinations (three biological replicates and three technical replicates for each extraction) were calculated and are given in mU g^-1^ (i.e. nmol substrate converted / product formed per minute and gram of wood dry mass).

Briefly, for analysis of fungal biomass, milled samples (0.5 g) were extracted with 25 ml of methanol as described by Newell et al. [39]. The methanol extracts were refluxed for 30 min at 70°C in the dark, saponified with 5 ml of 4% KOH in ethanol and extracted with hexane (10, 5, 5 ml) in a separator funnel. The combined hexane fractions were evaporated to dryness in a vacuum rotary evaporator. The residues were collected in 2 ml methanol, filtered (cellulose acetate, 0.45 lm) and stored in brown glass HPLC-vials at 2°C until analysis. Quantitative determination of ergosterol was conducted by reversed-phase HPLC; 20 μl of the extracts were injected by an HPLC autosampler (Beckmann Coulter, System Gold 125 Solvent Module) into a spherical silica-column (150 x 3 mm; MZ-Aquaperfect, MZ-Analysetechnik, Mainz). Methanol was used as the mobile phase at a flow rate of 0.5 ml min^-1^. Ergosterol was determined using a UV-detector (Beckmann Coulter, System Gold 166) at 282 nm and used for calculation of fungal biomass [10].

### Wood physico-chemical properties

To analyze the molecular mass distribution of aromatic lignocellulose fragments (water-soluble lignin fragments) formed as a consequence of fungal enzyme attack [40], a high-performance size exclusion chromatography (HPSEC) method was used with the water-extracted milled samples. The HPLC system (HP 1100 Liquid Chromatography, Hewlett-Packard, Waldbronn, Germany) was fitted with a HEMA-Bio linear column (8 × 300 mm, 10 mm) from Polymer Standard Service (Mainz, Germany); the same method was used by Noll et al. [10] and Arnstadt et al. [41]. The amounts of water-soluble lignin fragments and biomass, C and N, organic extractives, Klason lignin, acid-soluble lignin, water content and pH were calculated as reported previously in Noll et al. [10].

### Molecular fungal community analysis

#### DNA extraction, PCR and sequencing

Total community DNA was isolated from 0.25 g of each homogenized wood sample using a ZR Soil Microbe DNA MiniPrep kit (Zymo Research, Irvine, CA, USA) according to the manufacturer’s protocol. The quantity of genomic DNA was checked using a Nano Drop ND-1000 spectrophotometer (ThermoFisher Scientific, Dreieich, Germany). The fungal ITS2 region was amplified using a modified primer mix P7-3N-fITS7 and P7-4N-fITS7 (forward) together with P5-5N-ITS4 and P5-6N-ITS4 (reverse, see S1 Table), according to Ihrmark et al [42] and Hendgen et al [43]. PCR was performed in 25μl triplicate reactions, containing 12.5 μl of GoTaq Green Mastermix (Promega, Madison, USA), 25 μM of each primer and approximately 20 ng template DNA. Cycler conditions were as follows: denaturation period of 5 min at 95°C followed by 33 cycles of 95°C for 1 min, 55°C for 1 min, 72°C for 1 min 15 s and a final elongation step at 72°C for 10 min. After checking the quality of the PCR products by separation on a 1.5% agarose gel, the replicates were pooled and purified by gel extraction using an innuPREP Gel Extraction Kit (Analytik Jena, Jena, Germany). The purified DNA was quantified using a fluorescence spectrophotometer (Cary Eclipse, Agilent Technologies, Waldbronn, Germany). Subsequently, PCR products were sequenced with an Illumina MiSeq by the Deep Sequencing Group of the TU Dresden. Briefly, purified PCR products with universal 5’ tails were subjected to a second PCR of 6–8 cycles using Phusion HF (NEB) and two indexing primers: the P5 and the P7 primers (S1 Table). After indexing PCR, the final libraries were purified (1x XP Beads, Agencourt), equimolar pooled and used for 2 x 300 bp paired end sequencing on an Illumina MiSeq System.

#### Raw data, bioinformatic analysis, taxonomic and ecological assignment

Raw sequence data were imported and processed using Geneious R9 [44]. First, all forward and reverse reads were 5´ trimmed and adapter regions excluded. Then, forward and reverse reads were paired and the primer sequence was excluded. Next, the paired fungal sequences were quality trimmed using BBDuk (settings: trim low quality, minimum quality = 13) and merged to gather full length ITS2 gene regions using BBMerge (merge rate settings: very high) from BBTools. Generated sequences of 220 to 440 bp in length were exported for clustering and OTU table generation in SEED 2.0.4 [45]. There, the clustering based on USEARCH 8.1.1861 (32bit) included chimera removal, and species separation was based on 3% sequence dissimilarity [46]. This clustering uses a molecular species concept (operational taxonomic unit, OTU). However, the variability of the amplified short ITS sequence can potentially be based on technical PCR artifacts and also on intrinsic variability in a fungus, so that multiple OTUs could represent the same biological species. OTU tables were generated and the taxonomy was inferred by a blastn search against nr [46], and manually corrected using the MycoBank database. Ecological assignment followed Tedersoo et al. [47], and for wood-decomposing fungi Baldrian et al. [15], Arnstadt et al. [41] and Kriegelsteiner [48]. OTUs that comprised only singletons, doubletons and tripletons were not subject to further analyses. All processed and merged OTU sequences were submitted to the short read archive (SRA, https://www.ncbi.nlm.nih.gov/sra/) and are accessible under SRP102646.

### Statistics

Fungal OTU richness (hereafter referred to as species richness) was estimated for an identical set of sequences (11.501) using rarefaction integrated in PAST [49]. In five cases, the sequenced amount was below this threshold and extrapolation was carried out using a power function in MS Excel (R^2^ > 0.99). Multivariate analysis of the fungal community (OTUs) was performed without singletons, doubletons and tripletons. For all univariate statistics as well as multivariate statistics, wood parameters and enzyme activities were log transformed. All statistical analyses and calculations were conducted using R 3.0.3 [50]. For univariate statistics, normal distribution was tested using a Shapiro-Wilkinson-Test and Lilliefors-Test (modified Kolmogorov-Smirnov-Test, significance *p*<0.05) from the R package “nortest”. The effects of tree species, sapwood and heartwood, as well as deciduous and coniferous tree species on fungal species richness were analyzed by ANOVA using the “vegan” R package. Spearman rank correlations (rs, pairwise comparison) were calculated to examine relationships between selected wood parameters and enzyme activities with fungal species richness. Non-metric multidimensional scaling (NMDS) based on Bray-Curtis distances was conducted with the “vegan” package in R using the function “metaMDS” [51] to compare fungal community composition in the 13 temperate tree species. The influence of selected wood parameters (pH, Klason lignin, acid soluble lignin, organic extractives, biomass, water-soluble lignin fragments), enzyme activities and fungal family abundances on fungal community composition was analyzed by fitting data on each factor to the NMDS ordinations. The visualization of 3D-NMDS was achieved using the scatterplot 3D and “rgl” packages of R [52]. A goodness-of-fit-statistic (*R*^2^) for wood parameters and enzyme activities fitted to the NMDS ordinations was calculated using the *envfit* function with *P* values being based on 999 permutations [51]. All data are available in BExIS (https://www.bexis.uni-jena.de/PublicData/About.aspx) under numbers 24408, 24210 and 24209.

## Results

### Fungal community and ecology

**Fig 1:**
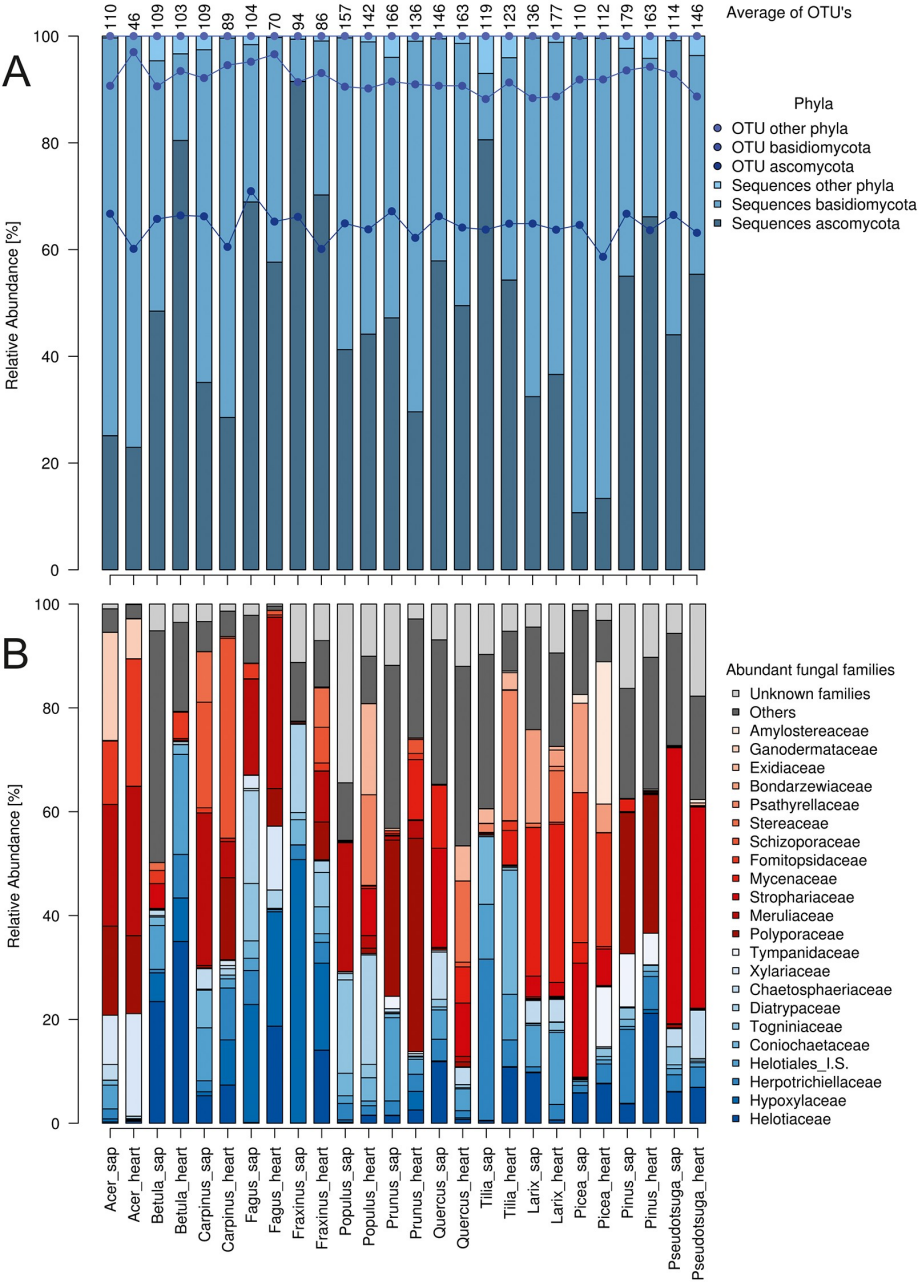
Fungal mean relative abundances. A) Sequence abundance (bars) and OTU affiliation (dots) with fungal phyla in 13 temperate European tree species in sapwood (sap) and heartwood (heart) (n = 3). Mean numbers of OTUs per tree sample are given at the top. B) Sequence abundance of the 22 most abundant fungal families (>20,000 sequences). Ascomycota are labeled in blue and Basidiomycota in red.

Altogether ~1.9 million sequences were used for clustering and resulted in ~3,000 OTUs. The removal of non-fungal sequences and singletons to tripletons finally resulted in 1,251 OTUs. After taxonomic evaluation, 61% of the OTUs were found to belong to the phylum Ascomycota and 26.4% to Basidiomycota, the remaining 12.6% to Mucoromycotina, Chytridiomycota and unknown taxa, with slightly different ratios in the collected CWD samples (Fig 1). In contrast, for relative abundances, 50.3% of the sequences affiliated with Basidiomycota and 47.8% with Ascomycota. However, we found marked differences in the relative sequence abundances of Ascomycota and Basidiomycota between the tree species and between sapwood and heartwood (Fig 1).

The dominant fungal class was Agaricomycetes (49.6% of all sequences) within the Basidiomycota; other abundant classes were Sordariomycetes, Leotiomycetes, Eurotiomycetes and Dothideomycetes (all Ascomycota). The most abundant families of the total of 194 detected were ascomycetous Helotiaceae (146,320 sequences) and basidiomycetous Polyporaceae (136,839 sequences), each with 7% relative sequence abundance. Altogether, we identified 22 fungal families with more than 20,000 sequences in each case (Fig 1).

The 3D-NMDS-ordination and corresponding goodness-of-fit-statistic (S1 Fig, S2 Table) revealed significant differences in the fungal community composition, particularly between tree species (*p* = 0.001, R^2^ = 0.5528), as well as between deciduous and coniferous wood (*p* = 0.001, R^2^ = 0.1844), but did not show significant differences between sapwood and heartwood when the entire dataset was considered (*p* = 0.834, R^2^ = 0.0035). Moreover, the community composition was not even significantly different between sapwood and heartwood when only tree species with a distinct heartwood fraction were analyzed (*p* = 0.897, R^2^ = 0.0053; i.e. *Fraxinus*, *Prunus*, *Quercus*, *Larix*, *Pinus*, *Pseudotsuga*).

The ordination revealed an association of fungi from the families Amylostereaceae (*Amylostereum* spp.), Bondarzewiaceae (*Heterobasidion* sp.), Mycenaceae (e.g. *Mycena* spp., *Sarcomyxa* sp.) and Strophariaceae (*Hypholoma capnoides*, *Galerina* sp., *Gymnopilus* sp.) mainly with coniferous tree species, while all other abundant families were associated with deciduous tree species (S1 Fig). For example, Meruliaceae (e.g. *Bjerkandera adusta*, *Phlebia* spp.) showed a clear tendency to colonize deciduous wood and were dominant in *Acer*, *Carpinus*, *Fagus* and in sapwood of *Populus*. The most abundant family, Helotiaceae (e.g. *Ascocoryne* spp.), was found in both coniferous and deciduous wood samples, and dominated logs of *Betula* and *Pinus* as well as the heartwood of *Fagus* and *Fraxinus* (Table 1). Furthermore, all 22 abundant fungal families established ‘starburst-like’ patterns of distribution in the NMDS, and there was no strong correlation between them (S1 Fig).

**Table 1:** List of the top 20 fungal species in this survey. Their occurrence and dominance in sapwood and heartwood is given, along with data pertaining to relative sequence abundances based on ITS2 sequences as well as their ecology. BRF, brown-rot fungus; SRF, soft-rot fungus; WRF, white-rot fungus; sap, saprotroph. A, Ascomycota; B, Basidiomycota; As, *Ascocoryne sarcoides*; Bj, *Bjerkandera adusta*; Le, *Leptodontidium* sp.; Hyph, *Hypholoma capnoides*; Co, *Coniochaeta* sp.; Hypox, *Hypoxylon rubiginosum*; Tr, *Trametes versicolor*; Fo, *Fomitopsis pinicola*; My, *Mycena* sp.; Sk, *Skeletocutis amorpha*; Cop, *Coprinellus micaceus*; Ph1, *Phialophora* sp.; Ph2, *Phialophora dancoi*; He, order Helotiales; Sch, *Schizopora radula*; Is, *Ischnoderma resinosum*; Phl, *Phlebia rufa*; Fom, *Fomes fomentarius*; Pha, *Phaeoacremonium hungaricum*; St, *Stereum sanguinolentum*.

Species	*As*	*Bj*	*Le*	*Hyph*	*Co*	*Hypox*	*Tr*	*Fo*	*My*	*Sk*	*Cop*	*Ph1*	*Ph2*	He	*Sch*	*Is*	*Phl*	*Fom*	*Pha*	*St*
Rank	1	2	3	4	5	6	7	8	9	10	11	12	13	14	15	16	17	18	19	20
**Ecology**	SRF	WRF	sap	WRF	SRF	SRF	WRF	BRF	WRF	WRF	sap	sap	sap		WRF	WRF	WRF	WRF		WRF
**Phylum**	A	B	A	B	A	A	B	B	B	B	B	A	A	A	B	B	B	B	A	B
**Total relative sequence abundance (%)**																				
	5.87	4.24	4.08	4.07	3.39	3.09	3.08	2.3	2.2	1.91	1.82	1.74	1.73	1.73	1.68	1.67	1.6	1.58	1.54	1.52
**Samples dominated by fungal species**																				
Sapwood	2	3	1	3	1	3	1	1	1	1		1	1	1	1	1	2	1	1	
Heartwood	5	2	1	1	1	1	2	1	1	1	2				1	1		1	1	1
*Acer*		1														2		2		
*Betula*	4																			
*Carpinus*		2								1					2					
*Fagus*	1	1				1											1			
*Fraxinus*						3													1	
*Populus*		1									1								1	
*Prunus*			1				3													
*Quercus*																				1
*Tilia*	1				2						1		1							
*Larix*	1		1						2											
*Picea*				1				2												
*Pinus*										1		1		1			1			
*Pseudotsuga*				3																
**Maximum relative sequence abundance (%)**																				
	51.88	79	50.48	87.88	68.77	67.85	89.84	87.43	91.3	75.46	75.25	30	48.41	29.82	60.98	98	55.18	68.18	53.19	43.11
**Samples with relative sequence abundance >10%**																				
	12	10	11	7	6	5	5	2	2	2	2	3	4	4	4	2	3	2	2	2

**Fig 2:**
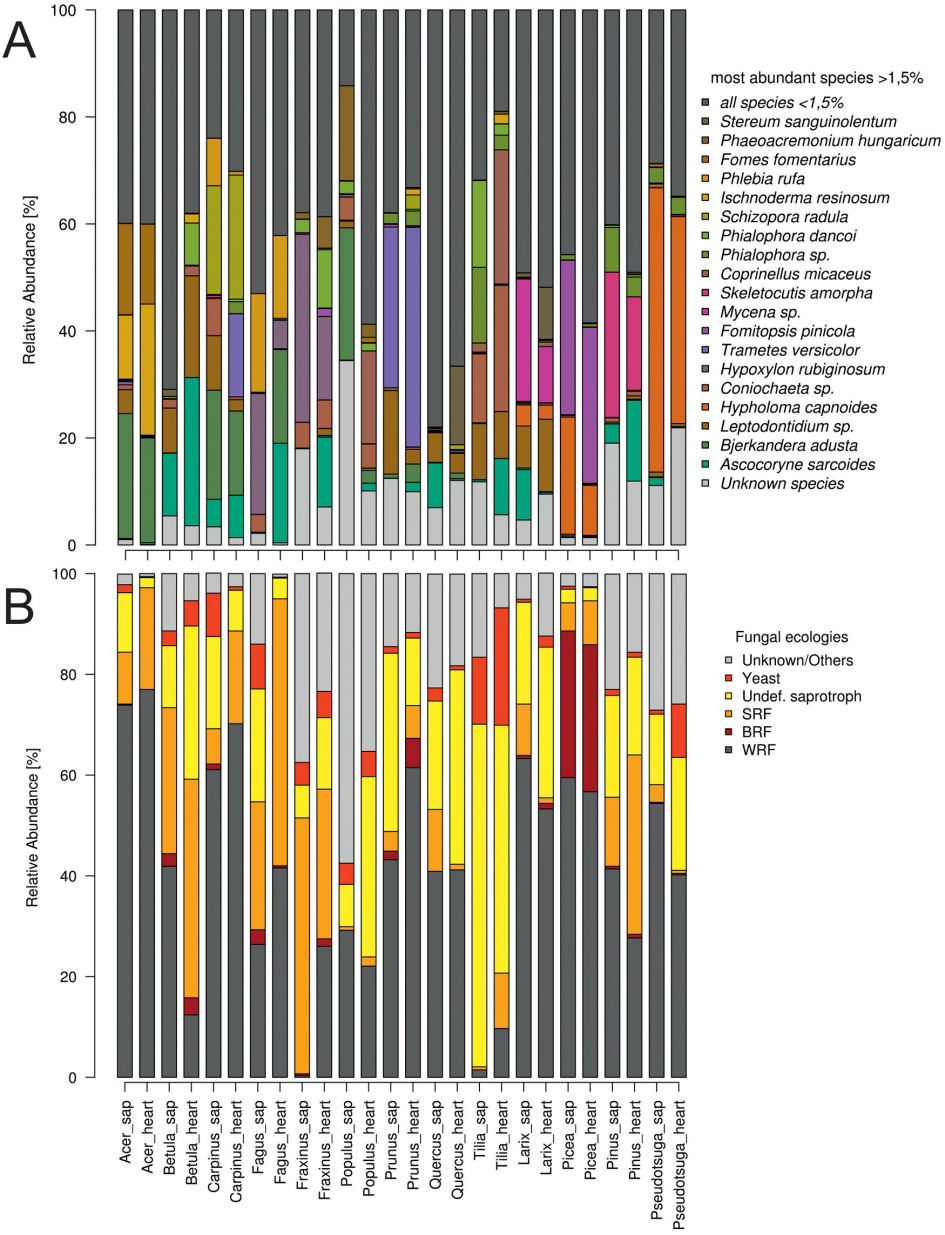
Mean relative sequence abundance of single fungal species (A) and different fungal ecotypes (B). The abundance is given for sapwood and heartwood of 13 European tree species (SRF—soft rot fungi, BRF—brown rot fungi, WRF—white rot fungi).

The most abundant fungal species was the ascomycete *Ascocoryne sarcoides*, with a sequence abundance of 5.87%, followed by the basidiomycete *Bjerkandera adusta* with 4.24% (Table 1). *A*. *sarcoides* was found in twelve samples with a relative sequence abundance higher than 10% and predominantly occurred in heartwood (less frequently in sapwood), particularly in decaying *Betula* logs (Table 1, Fig 2A). On the other hand, *B*. *adusta*, *Phlebia rufa*, *Hypholoma capnoides* and *Hypoxylon rubiginosum* were more abundant in sapwood than in heartwood (Table 1, Fig 2A); other species showed more uniform patterns in both wood types.

Altogether, the sequence abundance-based dataset comprised 43% white-rot fungi, 3% brown-rot fungi, 15% soft-rot fungi, 20% other saprotrophs (of undefined eco-physiological status, mainly Ascomycota), 4% yeasts and the remaining 15% included biotrophs, mycorrhizal fungi and species of unknown status.

Mean relative abundances of WRF ranged between 0.4% (sapwood of *Fraxinus*) and 77% (heartwood of *Acer*), for SRF between 0.6% (heartwood of *Pseudotsuga*) and 53% (heartwood of *Fagus*), for general undefined saprotrophs between 2% (heartwood of *Acer*) and 68% (sapwood of *Tilia*) and yeasts ranged from 0.2% (diverse heartwood samples) to 23.3% (heartwood of *Tilia* sp., mainly due to the presence of *Coniochaete* sp.). Brown-rot fungi were not recorded in heartwood or sapwood of *Populus*, *Quercus* and *Tilia*, nor in heartwood of *Acer* and *Carpinus*, but had a high relative abundance (29%) in heartwood and sapwood of *Picea* (Fig 2).

Based on the analysis of single tree logs, the highest relative abundances of WRF were found in heartwood samples of *Fagus* (99%), *Acer* (98.6%), *Picea* (98%) and *Quercus* (95.9%). BRF abundances were highest in samples of sapwood and heartwood of *Picea* (87.5% and 86.7%, respectively; mainly because of the occurrence of *Fomitopsis pinicola*), and SRF in sapwood and heartwood of *Fraxinus* (96.7% and 88.4%, high abundance of *Hypoxylon rubiginosum*) and *Fagus* (68% and 90.6%, respectively). The highest relative abundance of yeasts occurred in heartwood of a *Tilia* log with 67.4% (*Coniochaete* sp.) and *Pseudotsuga* with 30.9% (*Candida* sp.). *Phaeoacremonium hungaricum*, the most abundant classified biotroph, was found at a relative abundance of 53.2% in the sapwood of *Populus* (7.3% in heartwood) and heartwood of *Fraxinus* with 17.7% (Fig 2A).

### Fungal species richness and its relationship to extracellular enzymes and wood physicochemical parameters

**Fig 3:**
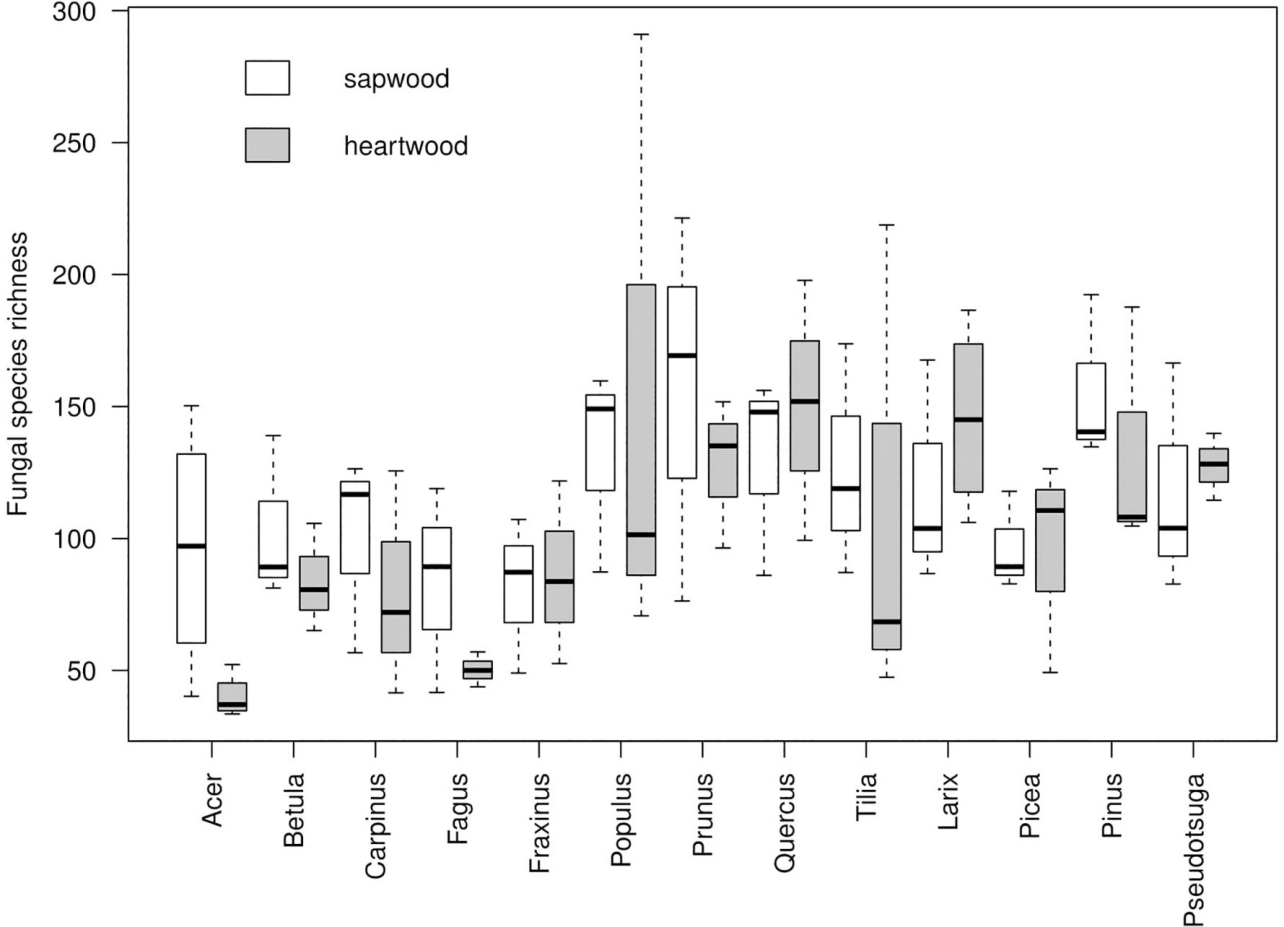
Fungal species richness. The richness is given for sapwood and heartwood samples of 13 decomposing temperate tree species.

Rarified fungal species richness varied between the decomposing tree samples from 33 (*Acer*) to 291 (*Populus*). The mean fungal species richness significantly differed between the tree species (*p* = 0.0019, ANOVA) and it was significantly higher in coniferous than deciduous trees (124 ± 36 *vs*. 103 ± 54, *p* = 0.0084) (Fig 3). It did not exhibit significant differences between sapwood and heartwood (114 ± 42 *vs*. 106 ± 56, *p* = 0.1099), even if only tree species with a distinct heartwood fraction (i.e. *Fraxinus*, *Prunus*, *Quercus*, *Larix*, *Pinus*, *Pseudotsuga*) were considered (125 ± 45 *vs*. 129 ± 38, *p* = 0.6345). The highest values of mean species richness were found in *Pinus* (145 ± 38), *Populus* (143 ± 80), *Prunus* (142 ± 52) and *Quercus* (140 ± 41), while low richness was recorded in *Carpinus* (90 ± 38), *Fraxinus* (83 ± 29), *Acer* (68 ± 43) and *Fagus* (67 ± 31) (Fig 3).

Extending a previous study [10], we analyzed five additional extracellular hydrolases (*β*-D-glucuronidase, *α*-D-mannosidase, *α*-L-arabinosidase, acid phosphatase, and sulfatase) involved in carbon, phosphorus and sulfur cycling (S2 Fig). All respective hydrolase activities exhibited significant differences between sapwood and heartwood (*p*< 0.05); in the case of phosphatase, glucuronidase, mannosidase and sulfatase, the activities were higher in sapwood than in heartwood. In deciduous trees, significantly higher activities were determined for phosphatase (*p*<0.01) and glucuronidase (*p*<0.05). In general, high mean values of enzyme activity were only observed for acid phosphatase in deciduous CWD (~37mU g^-1^ DM; S2 Fig), i.e. in sapwood of *Populus* and *Fraxinus* and in heartwood of *Carpinus* and *Tilia*.

**Fig 4:**
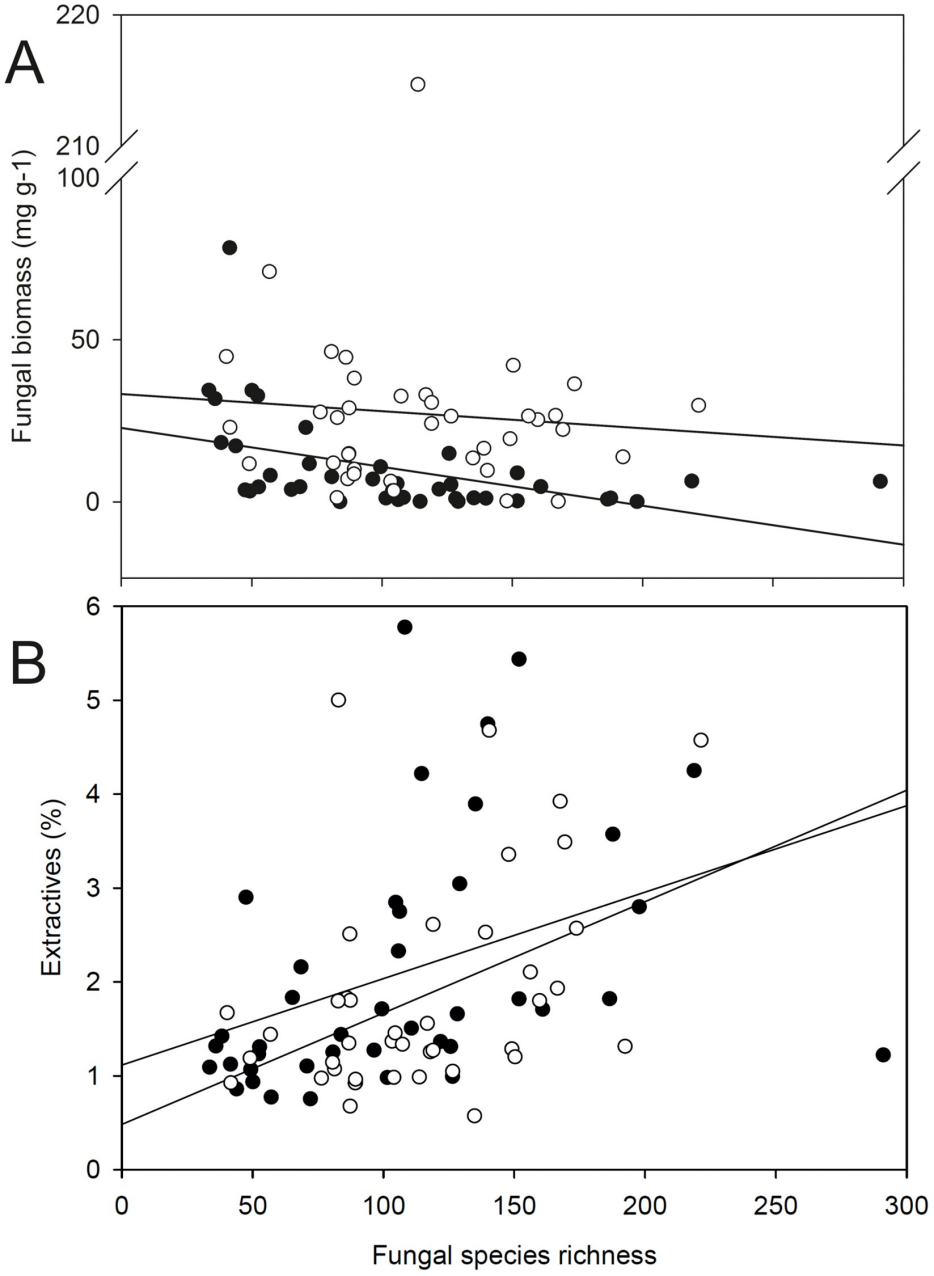
Correlation of fungal species richness with (A) fungal biomass and (B) wood extractives. White circles represent sapwood and black circles heartwood samples.

Spearman rank correlations for fungal species richness and enzyme activities revealed, in most cases, negative relationships for the 15 extracellular enzymes measured. For general peroxidase, manganese peroxidase, cellulase, xylanase, glucosidase and cellobiohydrolase, this effect was significant when the entire dataset was considered and it was even more pronounced when looking just at heartwood (S3 Table). Fungal species richness was also negatively correlated to fungal biomass, whilst it was positively correlated to organic extractives (Fig 4) and to the water content of sapwood (S3 Table).

### Fungal community composition in relation to extracellular enzymes and wood physicochemical parameters

**Fig 5:**
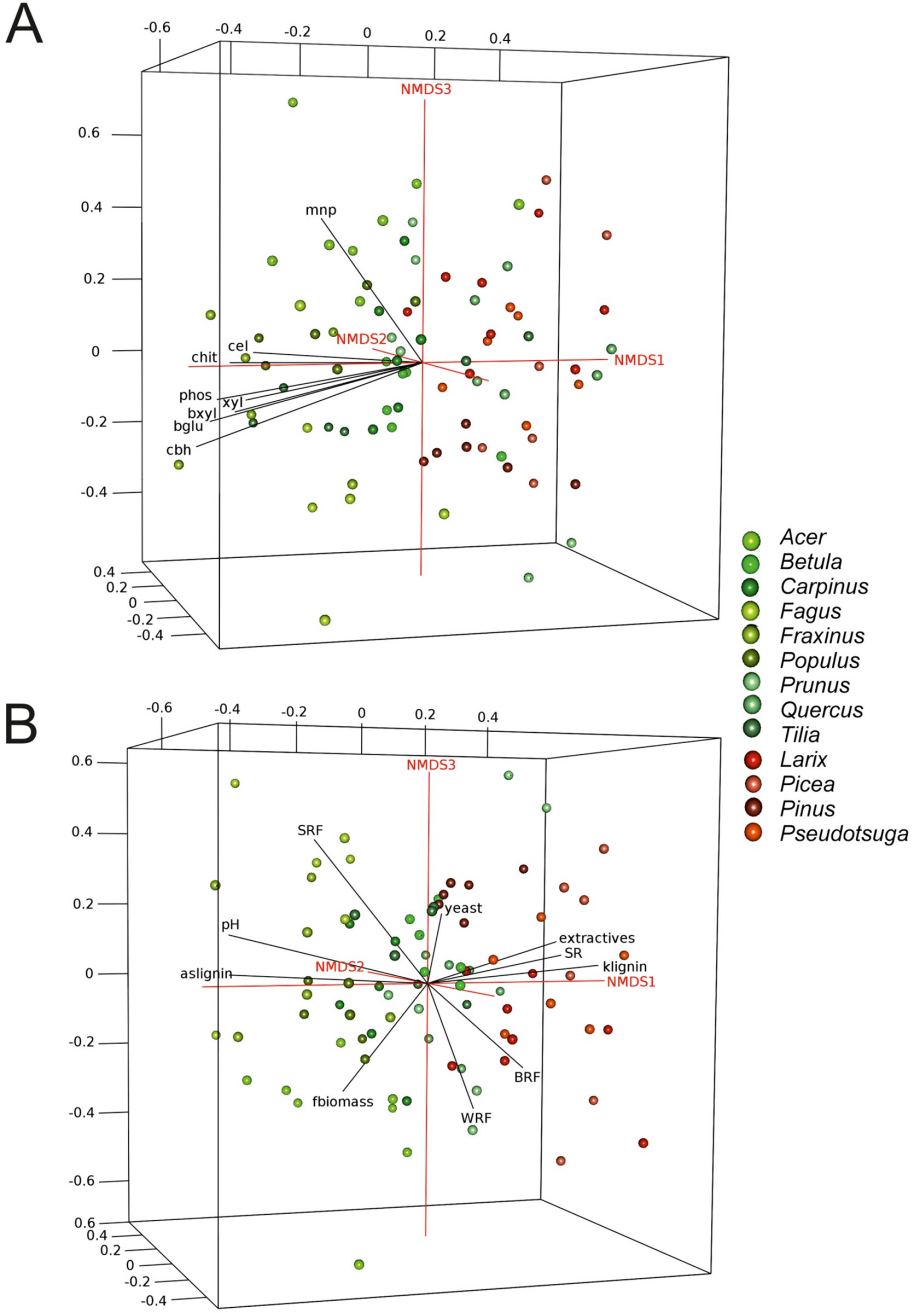
Non-metric multidimensional scaling (NMDS) ordination displaying fungal community composition. The ordination is given in relation to: A) fungal enzymes and B) wood parameters and abundances of ecotypes (green circles represent deciduous and red circles coniferous tree species). Abbreviations: WRF, white-rot fungi; BRF, brown-rot fungi; SRF, soft-rot fungi; aslignin, acid-soluble lignin; klignin, Klason-lignin; SR, species richness; mnp, manganese peroxidase; cel, cellulase; chit, chitinase; xyl, xylanase; bxyl, β-xylosidase; phos, acid phosphatase; bglu, β-glucosidase; cbh, cellobiohydrolase.

NMDS ordination and the goodness-of-fit-statistic revealed significant correlations between extracellular lignocellulolytic enzymes and the fungal community composition (Fig 5, S4 Table). Thus, lignocellulolytic enzymes, such as laccase, general peroxidase, manganese peroxidase, cellulase, xylanase, β-glucosidase, cellobiohydrolase, β-xylosidase, chitinase and acid phosphatase, were significantly correlated with the whole community, and in a similar manner, within sapwood and heartwood (S4 Table). In particular, high enzymatic activities correlated with the fungal community composition in deciduous tree species (Fig 5). Moreover, pH, Klason lignin, acid-soluble lignin, organic extractives, fungal biomass and fungal species richness were also significantly correlated with the fungal community composition. For example, higher Klason lignin content correlated with communities in coniferous tree species, while acid-soluble lignin did so with communities in deciduous tree species (Fig 5). The fungal ecotypes (WRF, SRF, BRF and ‘yeast’) were significantly correlated to different tree species (S4 Table, Fig 5). For example, higher species richness and relative abundances of BRF were associated with communities in coniferous tree species, whereas many of the other parameters were related to the fungal community of deciduous tree species (Fig 5). In contrast, yeasts (mainly ascomycetous *Coniochaeta* spp.) and WRF appeared to be correlated with different deciduous and coniferous tree species (Fig 5). No or just weak correlations were found for water-soluble lignin fragments and the total N or water content (S4 Table).

### Fungal family and eco-type abundances in relation to measured extracellular enzymes

**Fig 6:**
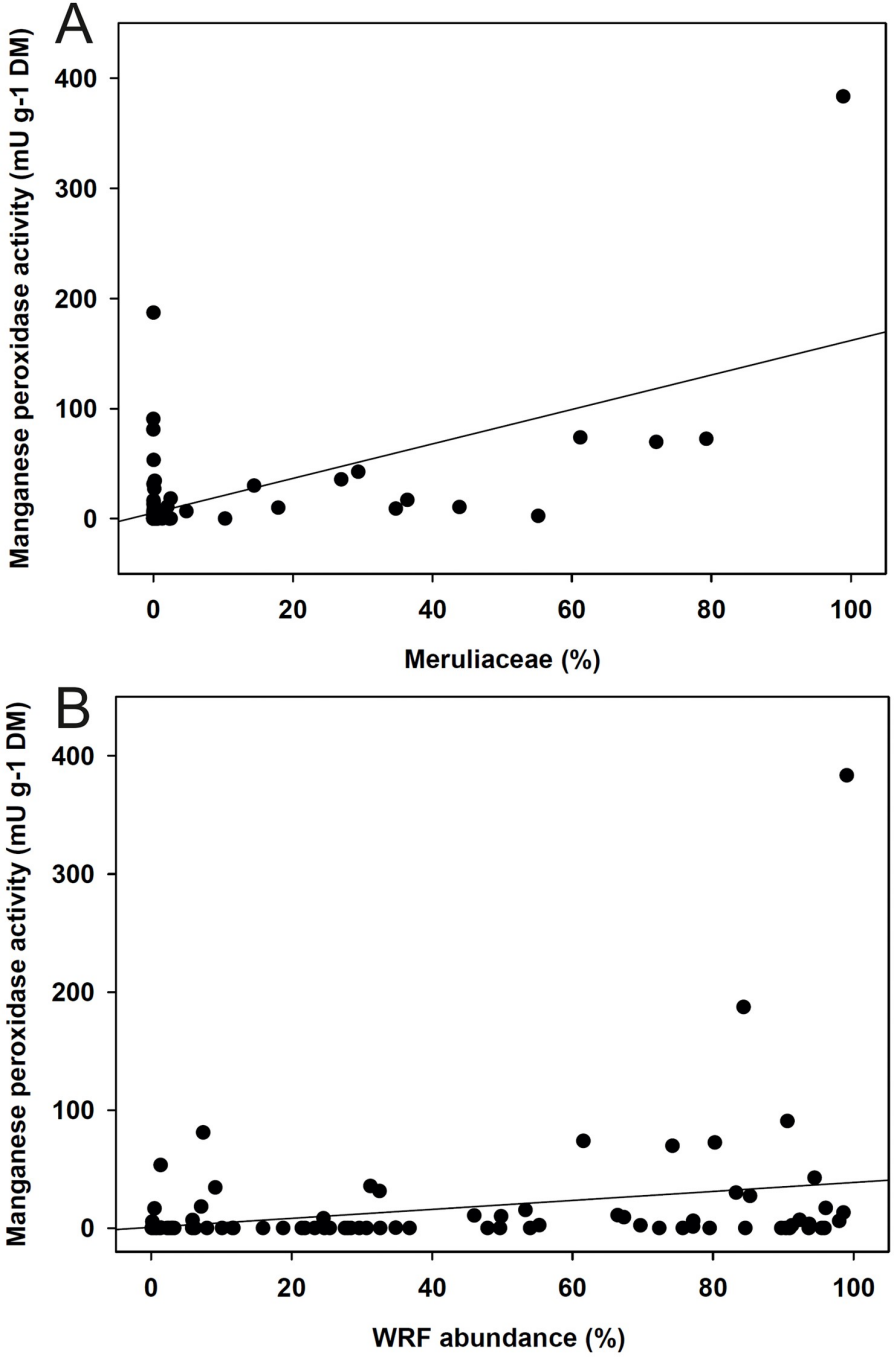
Results of correlations. The correlation of the relative sequence abundance for A) Meruliaceae (ϱ = 0.26) and B) all white-rot fungi to extracellular manganese peroxidase activity (ϱ = 0.29) measured in the wood samples.

Significant positive correlations were found for Meruliaceae and WRF with ligninolytic enzyme activities of manganese peroxidase and general peroxidase (p < 0.05; S5 Table, Fig 6). In more detail, WRF showed a significant positive correlation with manganese peroxidase when considering only deciduous tree species (p = 0.002, ρ = 0.41) or only heartwood (p = 0.048, ρ = 0.31). Furthermore, Coniochaetaceae showed significant positive correlations with laccase and different (hemi)cellulolytic enzymes (e.g. cellobiohydrolase). In a few cases, the abundances of Hypoxylaceae (e.g. β-glucosidase), Diatrypaceae, Schizoporaceae and Psathyrellaceae were also positively correlated with different hydrolases (S5 Table).

## Discussion

In the present study, we analyzed the molecular fungal community composition and species richness intensively to understand differences in extracellular enzyme activities and wood decomposition. The recognized 1,251 different fungal OTUs in our experiment (excluding rare OTUs, i.e. singletons to tripletons), with a mean richness of 110 ± 49 per sample are in the range of other European studies using 454 pyrosequencing (mean richness in the range ~20 to 50 for similar experiments; [5,16,31,53] and current Illumina MiSeq NGS approaches (~200 to 250) [15]. It should be noted, however, that such numbers are strongly dependent on the sequencing depth and the bioinformatic pipeline used subsequently, e.g. the clustering algorithms and removal of rare OTUs. They are also dependent on the analyzed wood decay class, as—in general—the richness appears to increase as decay proceeds [42,53,54], and our experiment can still be regarded as only covering an early phase after 6 years of tree log exposure on the ground. Nevertheless, higher richness and detection of rarer taxa can be expected with deeper sequencing of the wood samples. Purahong et al. [31] detected a total of 1,254 OTUs and mean richness between 22 and 42 OTUs per log in a total of 297 logs representing eleven tree species and a sequencing depth of ~3,000 per sample (454 approach from 2012 in the BELongDead experiment). In our 2014 experiment, using a subset of 41 logs from the BELongDead experiment, we found a similar number of OTUs (1,150 without singletons to quadrupletons, comparable to Purahong et al. [31]), but with a considerably higher sequencing depth (~11,500 sequences per sample) and using slightly different primer regions. Thus in upscaling of this NGS technique to the full scale of the BELongDead Experiment (>1,100 logs), we expect even more fungal OTUs to be revealed.

As expected in hypothesis 1 (H1), and in accordance with a previous molecular survey [31] and sporocarp surveys [55,56], our study revealed significant tree species effects for the fungal species richness and community composition, and also between decomposing deciduous and coniferous tree species. This is likewise in accordance with another molecular study involving three different tree species over longer decomposition times [15], and can be explained by different wood-chemical characteristics and/or the adaption of the fungal community and its decomposition machinery to this (e.g. *via* different types of wood-rot). On the other hand, unexpectedly neither significant differences in fungal species richness nor community composition were observed between sapwood and heartwood of the 13 tree species (H2). Information about differences in the molecular fungal community between sapwood and heartwood are rather scarce, not showing clear tendencies and involving only a few tree species [5,26]. In the present study, we did not find differences in the fungal communities when tree species with distinct heartwood fractions were considered (i.e. *Fraxinus excelsior*, *Prunus avium*, *Quercus* sp., *Larix decidua*, *Pinus sylvestris*, *Pseudotsuga menziesii*). After six years of decomposition, obviously, fungal species fully occupied the decaying logs. However, there were largely differing amounts of biomass and concomitant enzyme activities, i.e. higher in sapwood than in heartwood [10]. The occurrence of distinct heartwood negatively correlated with the decay rate of the 13 tree species [11]. Heartwood is known to contain higher amounts of secondary metabolites (phenolics, tannins, stilbenes) than sapwood, which could hamper the growth of fungi [57]. However, it did not result in a significant separation of distinct community compositions between sapwood and heartwood.

In only a few cases, one fungal species were predominantly found either in sapwood or heartwood, for example, *Ascocoryne sarcoides* in heartwood or *Phlebia rufa* and *Hypholoma capnoides* in sapwood. Ascomycetous *A*. *sarcoides* generally dominated in this experiment, and it is one fungus specifically known to grow in heartwood (associated with the ‘heart-rot’ of logs) [26] and is even endophytic prior to tree death [58]. Moreover, it has been reported that this fungus is a competitive cellulose decomposer with antibacterial activity, well adapted to this environment [59,60]. The second fungus generally dominant in the logs in our study was *B*. *adusta*, a basidiomycete known to cause severe white-rot and belonging to the Meruliaceae. *B*. *adusta* and other species of this family (e.g. *Phlebia* spp., *Phanerochaete* spp.) are known to produce diverse ligninolytic peroxidases, including different manganese peroxidases (MnPs, EC 1.11.1.13), which all belong to the class II peroxidase family [60–63]. In this study, we found a significant correlation between the sequence abundance of Meruliaceae and the extracellular activities of manganese peroxidases. Thus, this finding provides strong ‘*in-situ* evidence’ for the importance of this fungal family that efficiently uses MnPs to overcome the lignin barrier in wood (H4) [40]. Further this correlation was also found when considering the abundance of all WRF. However, such a relationship was not found for the abundance of white-rot families (Basidiomycota) and (hemi)cellulolytic enzyme activities, despite the fact that WRF have plenty of these enzymes available in their genomes [19]. On the other hand, the abundance of the ascomycetous family Coniochaetaceae correlated well with extracellular activities of laccase and different (hemi)cellulolytic enzymes, as well as that of Hypoxylaceae to *β*-D-glucosidase, thus providing ‘field evidence’ for their importance in wood decay. Not least, recent sequencing of the genome of *Coniochaeta hoffmannii* revealed the presence of several lignocellulolytic enzymes and additionally supports their active role in wood decomposition [64]. Recently, Hori and coworkers [65], could identify many species actively expressing lignocellulolytic enzymes during decomposition of *Pinus contorta* logs using an omics approach.

In the present study, numerous extracellular enzymes involved in element mobilization (carbon, nitrogen, phosphorus) showed significant relationships with the fungal community composition, as was similarly observed in a recent study dealing with deadwood decomposition in a temperate forest [15]. They furthermore reveal a similar and well-known spectrum of fungal groups in general (for example, with respect to richness and relative abundance of Ascomycota *vs*. Basidiomycota) and wood-decomposing fungal species in particular (with similar relative abundances of fungal life styles, i.e. high proportion of white-rot *vs*. brown-rot fungi). The white-rot mechanism seemed to dominate in this study, but with differences among tree species and between sapwood and heartwood. *Tilia* logs, for example, were instead colonized by undefined saprotrophs or yeasts. The sapwood of *Fraxinus*, as another example, revealed a high relative abundance of *Hypoxylon rubiginosum*, which is a SRF known to be capable of producing robust melanized crusts, which could prevent other fungi from colonization. However, the strong wood decomposition at least in many of the decomposing tree species is driven by WRF, which in the evolution of the Agaricomycetes is the ancestral ecology and thus widespread among this fungal class and well adapted to this environment [66]. Physicochemical wood parameters such as pH, lignin and extractives significantly correlated with the fungal community as well, and hence may explain its structure, as also seen in other recent studies [16,67] and points to an active fungal control and transformation of woody micro-environments.

However, correlation analyses involving fungal species richness also revealed unexpected results. In particular, the activities of extracellular ligninolytic and (hemi)cellulolytic enzymes showed negative correlations with species richness. A similar finding was previously reported for ligninolytic enzymes [16] and can be explained by the negative correlation between fungal biomass and species richness, which is contrary to our third hypothesis (H3). It seems that in this study, biomass is accumulated by a few dominant species, i.e. white-rot or soft-rot species that markedly promote enzyme and/or chemical radical production and hence wood decomposition. Conversely, higher species richness may force species interactions, including competition and antagonistic ‘behavior’ and thus, might inhibit the increase in fungal biomass. It has been shown that enhanced competition for space, resources or nutrients in species-rich communities can have a negative impact on the rate of wood decomposition, since it might reduce mycelial growth or fungal activity [30,68]. Other studies have demonstrated that during fungus-to-fungus interactions, certain enzyme activities can increase, for example, that of laccase in confronting basidiomycetes (i.e. [69]). In our present study, however, we did not find any indication of an increase in laccase activity with increasing fungal species richness.

Unexpectedly, a higher species richness negatively correlated with fungal biomass and positively correlated with higher content of extractives, although these compounds, such as resins, terpenoids and phenolics, are known to inhibit fungal growth or the secretion of particular enzymes [54]. In the case of high species richness, we expect that multiple fungi had tried to establish in logs with high extractive content but with limited success. Thereby these fungi developed no significant biomass over six years of decomposition, but were still detectable in molecular analyses.

Due to the variety of the deadwood tree species investigated and the discrimination between sapwood and heartwood, the present study provides a particularly detailed view on the molecular diversity of wood-inhabiting fungi. It links specific fungal groups to distinct enzymatic activities and thus gives ‘*in-situ* evidence’ for their contribution to the decomposition of deadwood. Overall, this improves our understanding of the complex fungal-mediated ecosystem processes going on during wood decay.

## Supporting information

S1 Fig 3D non-metric multidimensional scaling (3D-NMDS). NMDS ordination of the most abundant fungal families colonizing 13 temperate European tree species.
(TIFF)

S2 Fig Overview of measured enzyme activities. The activities are given for samples **from** 13 temperate European tree species.
(TIFF)

S1 Table Sequences of the primers used in this study. (PDF)

S2 Table Result of the goodness-of-fit-statistic (R2). The results are given for the abundant fungal families against the 3D-NMDS ordination of the fungal OTUs for all samples as well as for sapwood and heartwood. Shaded in grey: significance (uncorrected) p <0.05.
(PDF)

S3 Table Spearman rank correlations (*p*-value and Rho (*ϱ*)) of fungal species richness with enzyme activities and wood parameters. Comparing all samples or sapwood and heartwood separately; Significance p <0.05 (uncorrected) is indicated by grey-shading.
(PDF)

S4 Table Goodness-of-fit-statistic (R2). The results are given for enzyme activities and wood parameters against the 3D-NMDS ordination of the fungal OTUs for all samples as well as for sapwood and heartwood. Shaded in grey: significance (uncorrected) p <0.05.
(PDF)

S5 Table Spearman rank correlations. Results are given for the most abundant fungal families and ecotypes to the measured extracellular enzymes. Shaded, significance p < 0.05 (uncorrected).
(PDF)
