# Supplementary figures and images for: Molecular fungal community and its decomposition activity in sapwood and heartwood of 13 temperate European tree species
Source: PLoS One. 2019 Feb 14;14(2):e0212120. doi: 10.1371/journal.pone.0212120 (PMC6375594; doi:10.1371/journal.pone.0212120)

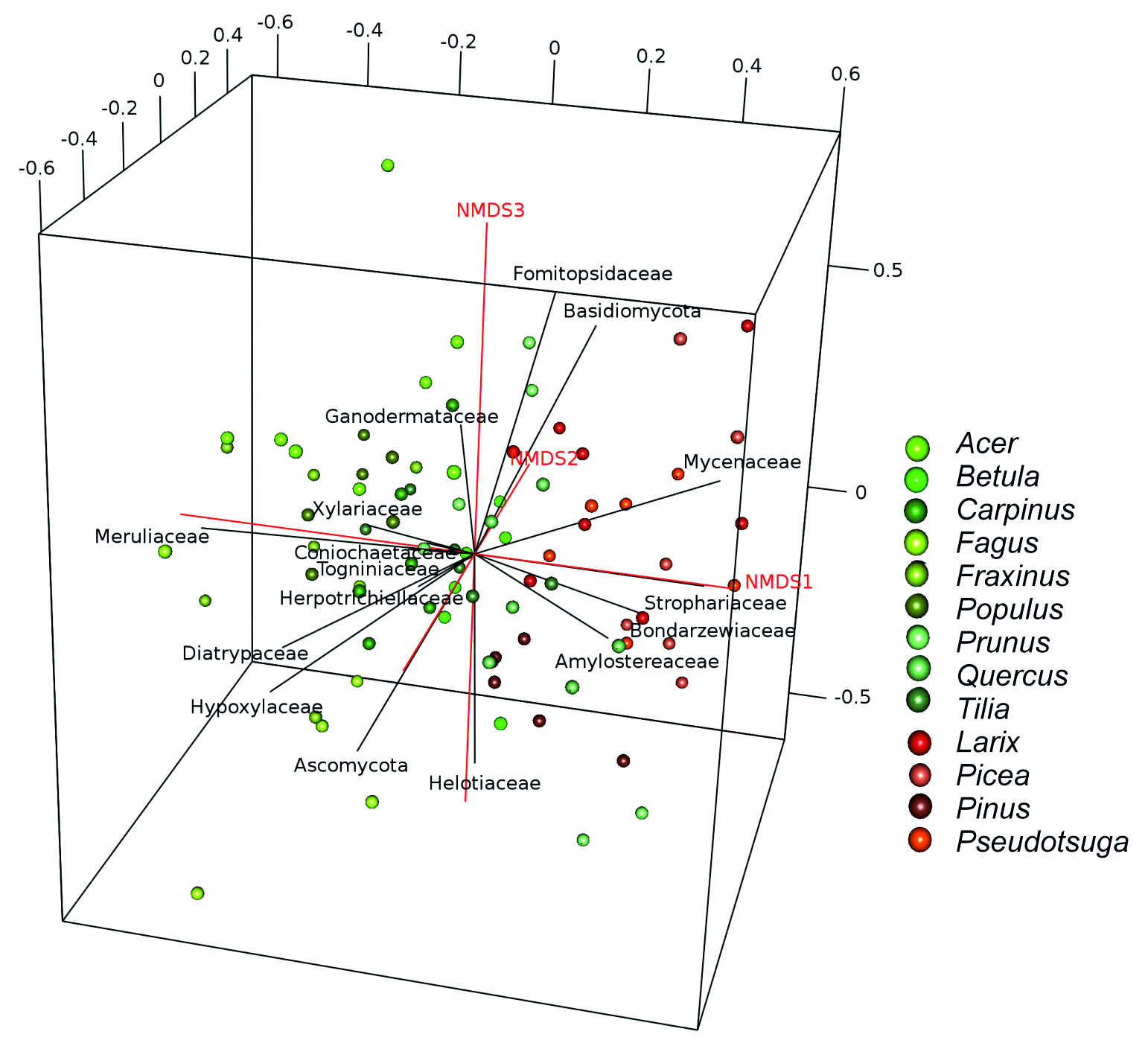

Supplement: S1 Fig — NMDS ordination of the most abundant fungal families colonizing 13 temperate European tree species. (TIFF) [file pone.0212120.s001.tiff]

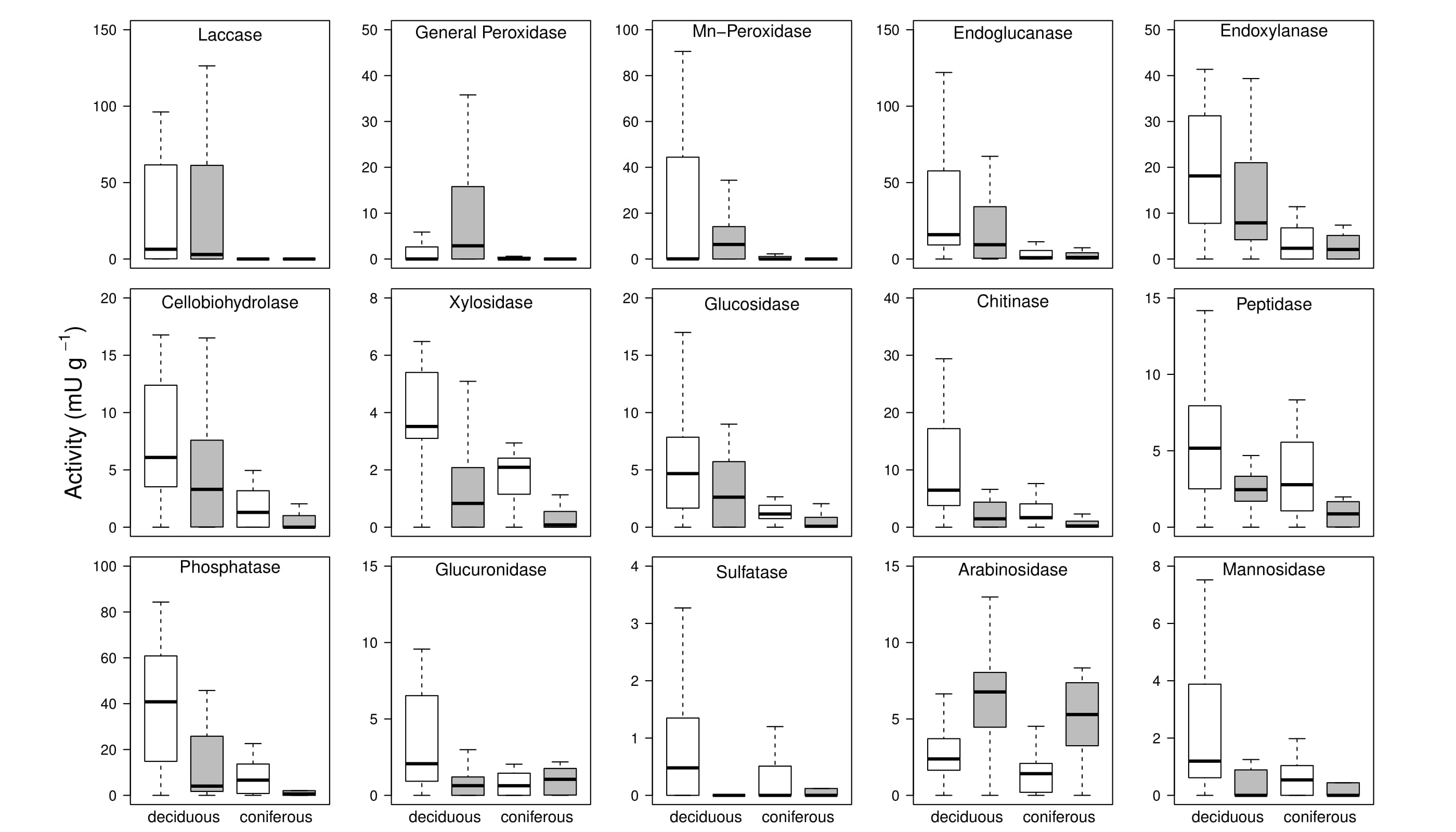

Supplement: S2 Fig — The activities are given for samples from 13 temperate European tree species. (TIFF) [file pone.0212120.s002.tiff]
