# Supplementary material for: Molecular fungal community and its decomposition activity in sapwood and heartwood of 13 temperate European tree species
Source: PLoS One. 2019 Feb 14;14(2):e0212120. doi: 10.1371/journal.pone.0212120 (PMC6375594; doi:10.1371/journal.pone.0212120)
Supplement: S1 Table — (PDF) [file pone.0212120.s003.pdf]

Name	Primer 5´-3´
P5_5N_ITS4	ACACTCTTTCCCTACACGACGCTCTTCCGATCTNNNNNTCCTCCGCTTATTGATATGC
P5_6N_ITS4	ACACTCTTTCCCTACACGACGCTCTTCCGATCTNNNNNNTCCTCCGCTTATTGATATGC
P7_3N_fITS7	GTGACTGGAGTTCAGACGTGTGCTCTTCCGATCTNNNGTGARTCATCGAATCTTTG
P7_4N_fITS7	GTGACTGGAGTTCAGACGTGTGCTCTTCCGATCTNNNNGTGARTCATCGAATCTTTG
P5-index	AATGATACGGCGACCACCGAGATCTACACiiiiiiiACACTCTTTCCCTACACGACGCTCTTCCGATC*T
P7-index	CAAGCAGAAGACGGCATACGAGATiiiiiiiiGTGACTGGAGTTCAGACGTGTGCTCTTCCGATC*T
