# Supplementary material for: Molecular fungal community and its decomposition activity in sapwood and heartwood of 13 temperate European tree species
Source: PLoS One. 2019 Feb 14;14(2):e0212120. doi: 10.1371/journal.pone.0212120 (PMC6375594; doi:10.1371/journal.pone.0212120)
Supplement: S2 Table — The results are given for the abundant fungal families against the 3D-NMDS ordination of the fungal OTUs for all samples as well as for sapwood and heartwood. Shaded in grey: significance (uncorrected) p <0.05. (PDF) [file pone.0212120.s004.pdf]

	Total		Sapwood		Heartwood	
Abundant families	R^2	*P*	R^2	*P*	R^2	*P*
Meruliaceae	0.3216	**0.001 *** **	0.0332	0.742	0.4758	**0.001 *** **
Coniochaetaceae	0.1735	**0.002 ** **	0.1517	0.120	0.1935	**0.028 * **
Herpotrichiellaceae	0.1568	**0.002 ** **	0.2750	**0.022 * **	0.1930	**0.049 * **
Xylariaceae	0.1751	**0.003 ** **	0.2682	**0.012 * **	0.3383	**0.001 *** **
Tricholomataceae	0.1532	**0.005 ** **	0.1966	0.052 .	0.1716	0.059 .
Strophariaceae	0.1436	**0.005 ** **	0.1136	0.187	0.1791	**0.049 * **
Helotiaceae	0.1573	**0.006 ** **	0.2189	**0.021 * **	0.1803	0.051 .
Fomitopsidaceae	0.1228	**0.014 * **	0.1722	0.080 .	0.1318	0.150
Diatrypaceae	0.1194	**0.020 * **	0.3003	**0.006 ** **	0.0719	0.439
Polyporaceae	0.1164	**0.025 * **	0.0549	0.514	0.2193	**0.023 * **
Togniniaceae	0.0993	**0.039 * **	0.1210	0.169	0.0937	0.334
Ganodermataceae	0.0823	0.067 .	0.0992	0.229	0.1549	0.056 .
Bondarzewiaceae	0.0874	0.068 .	0.1397	0.133	0.1144	0.230
Exidiaceae	0.0817	0.091 .	0.0576	0.476	0.1671	**0.044 * **
