# Supplementary material for: Molecular fungal community and its decomposition activity in sapwood and heartwood of 13 temperate European tree species
Source: PLoS One. 2019 Feb 14;14(2):e0212120. doi: 10.1371/journal.pone.0212120 (PMC6375594; doi:10.1371/journal.pone.0212120)
Supplement: S3 Table — Comparing all samples or sapwood and heartwood separately; Significance p <0.05 (uncorrected) is indicated by grey-shading. (PDF) [file pone.0212120.s005.pdf]

	**Total**		**Sapwood**		**Heartwood**	
	P	*ϱ*	*P*	*ϱ*	*P*	*ϱ*
**Enzyme activity**						
Laccase	0.436	-0.087	0.496	0.109	0.146	-0.231
General peroxidase	0.008	-0.290	0.959	0.008	0.002	-0.467
Manganese peroxidase	0.014	-0.271	0.721	-0.057	0.0004	-0.529
Endo-1,4-*ß*-cellulase	0.003	-0.328	0.320	-0.159	0.0002	-0.550
Endo-1,4-*ß*-xylanase	0.001	-0.356	0.167	-0.220	0.0003	-0.537
ß-D-glucosidase	0.0002	-0.396	0.067	-0.289	0.0008	-0.505
Cellobiohydrolase	0.004	-0.318	0.349	-0.150	0.0007	-0.509
ß-D-xylosidase	0.056	-0.212	0.066	-0.290	0.042	-0.319
ß-N-acetylhexosaminidase (Chitinase)	0.065	-0.205	0.542	-0.098	0.008	-0.410
Leucine-aminopeptidase (Peptidase)	0.186	-0.147	0.842	-0.032	0.012	-0.389
ß-D-glucuronidase	0.726	-0.039	0.974	-0.005	0.266	-0.178
α-D-mannosidase	0.703	-0.043	0.518	-0.104	0.672	0.068
α-L-arabinosidase	0.090	-0.188	0.716	-0.059	0.027	-0.345
Acid phosphatase	0.916	-0.012	0.509	0.106	0.137	-0.236
Sulfatase	0.372	0.100	0.916	0.017	0.864	0.028
**Wood parameter**						
pH	0.106	-0.180	0.500	-0.108	0.478	-0.114
Klason lignin (%)	0.069	0.202	0.433	0.126	0.153	0.227
Acid soluble lignin (%)	0.107	-0.179	0.676	-0.067	0.192	-0.208
Organic extractives (%)	0.00001	0.466	0.010	0.396	0.0003	0.539
Water soluble lignin fragments (mg g^{-1})	0.415	-0.091	0.268	0.177	0.002	-0.466
Fungal biomass (mg g^{-1})	0.017	-0.262	0.658	-0.071	0.0001	-0.570
Total N (%)	0.504	-0.075	0.439	0.124	0.005	-0.431
Water content (g g^{-1})	0.088	0.190	0.034	0.332	0.820	-0.037
