# Supplementary material for: Molecular fungal community and its decomposition activity in sapwood and heartwood of 13 temperate European tree species
Source: PLoS One. 2019 Feb 14;14(2):e0212120. doi: 10.1371/journal.pone.0212120 (PMC6375594; doi:10.1371/journal.pone.0212120)
Supplement: S4 Table — The results are given for enzyme activities and wood parameters against the 3D-NMDS ordination of the fungal OTUs for all samples as well as for sapwood and heartwood. Shaded in grey: significance (uncorrected) p <0.05. (PDF) [file pone.0212120.s006.pdf]

	Total		Sapwood		Heartwood	
	R^2	*P*	R^2	*P*	R^2	*P*
**Enzyme activity**						
Laccase	0.0694	0.126	0.1726	0.070 .	0.1889	0.048 *
General peroxidase	0.1373	0.007 **	0.0531	0.571	0.2355	0.017 *
Manganese peroxidase	0.2632	0.001 ***	0.1346	0.140	0.3099	0.007 **
Endo-1,4-ß-cellulase	0.1288	0.023 *	0.2425	0.014 *	0.1449	0.125
Endo-1,4-ß-xylanase	0.2814	0.001 ***	0.3086	0.004 **	0.3467	0.003 **
ß-D-glucosidase	0.3247	0.001 ***	0.4170	0.002 **	0.2999	0.005 **
Cellobiohydrolase	0.3690	0.001 ***	0.4796	0.001 ***	0.3727	0.001 ***
ß-D-xylosidase	0.1390	0.006 **	0.0733	0.393	0.1766	0.057 .
ß-N-acetylhexosaminidase (Chitinase)	0.2305	0.001 ***	0.2799	0.006 **	0.3999	0.001 ***
Leucine-aminopeptidase (Peptidase)	0.1040	0.039 *	0.0668	0.469	0.1913	0.059 .
ß-D-glucuronidase	0.0767	0.104	0.2376	0.022 *	0.0377	0.685
α-D-mannosidase	0.0378	0.384	0.2018	0.035 *	0.0594	0.536
α-L-arabinosidase	0.0343	0.431	0.1149	0.217	0.0516	0.594
Acid phosphatase	0.1313	0.014 *	0.1920	0.042 *	0.2300	0.021 *
Sulfatase	0.0633	0.169	0.1442	0.118	0.0647	0.472
**Wood parameter**						
pH	0.4538	0.001 ***	0.4554	0.002 **	0.6030	0.001 ***
Klason lignin (%)	0.1617	0.005 **	0.1616	0.095 .	0.2937	0.004 **
Acid-soluble lignin (%)	0.3961	0.001 ***	0.3057	0.004 **	0.5120	0.001 ***
Organic extractives (%)	0.1267	0.019 *	0.0492	0.596	0.2230	0.020 *
Water-soluble lignin fragments (mg g^{-1})	0.0642	0.170	0.0539	0.586	0.1990	0.037 *
Fungal biomass (mg g^{-1}) (deduced from ergosterol content)	0.1406	0.008 **	0.1793	0.071 .	0.2595	0.011 *
Total N (%)	0.0464	0.292	0.1626	0.078 .	0.1405	0.131
Species richness	0.2123	0.001 ***	0.2509	0.009 **	0.2989	0.003 **
Water content (g g^{-1})	0.0393	0.379	0.1325	0.152	0.0790	0.392
**Eco-types**						
WRF	0.1579	0.004 **	0.2961	0.008 **	0.0087	0.947
BRF	0.1156	0.026 *	0.1715	0.075	0.1687	0.047 *
SRF	0.2305	0.001 ***	0.0882	0.310	0.4599	0.001 ***
Yeast	0.1649	0.004 **	0.1645	0.081	0.1792	0.055
