# Supplementary material for: Molecular fungal community and its decomposition activity in sapwood and heartwood of 13 temperate European tree species
Source: PLoS One. 2019 Feb 14;14(2):e0212120. doi: 10.1371/journal.pone.0212120 (PMC6375594; doi:10.1371/journal.pone.0212120)
Supplement: S5 Table — Results are given for the most abundant fungal families and ecotypes to the measured extracellular enzymes. Shaded, significance p < 0.05 (uncorrected). (PDF) [file pone.0212120.s007.pdf]

most abundant families	Laccase		General peroxidase		Manganese peroxidase		Endo-1,4-ß-cellulase		Endo-1,4-ß-xylanase		ß-D-glucosidase		Cellobio-hydrolase		ß-D-xylosidase	
	p	ϱ	p	ϱ	p	ϱ	p	ϱ	p	ϱ	p	ϱ	p	ϱ	p	ϱ
Meruliaceae	0.4199	0.0902	**0.0072**	**0.0246**	**0.0174**	**0.2621**	0.3879	0.0966	0.2786	0.1211	0.3993	0.0943	0.0938	0.1863	0.4482	0.0849
Coniochaetaceae	**0.0159**	**0.2655**	0.5019	-0.0752	0.9081	0.0129	0.4428	0.0859	**0.0167**	**0.2635**	**0.0050**	**0.3071**	**0.0047**	**0.3091**	0.0516	0.2157
Herpotrichiellaceae	0.1274	0.1697	0.0538	-0.2137	0.1087	-0.1785	0.5067	-0.0744	0.6375	-0.0528	0.9329	-0.0094	0.6131	0.0567	0.3559	0.1032
Xylariaceae	0.7097	0.0417	0.8773	0.0173	0.4756	0.0799	0.6013	0.0586	0.4470	0.0851	0.5868	0.0609	0.6005	-0.0587	0.1348	-0.1665
Hypoxylaceae	0.8228	-0.0251	0.2534	0.1276	0.2743	0.1222	0.3520	0.1041	0.1973	0.1438	**0.0491**	**0.2180**	0.0917	0.1875	0.2347	0.1337
Strophariaceae	0.0742	-0.1982	0.2988	-0.1161	0.2661	-0.1242	0.0356	-0.2345	0.0002	-0.3963	0.0010	-0.3579	0.0205	-0.2555	0.2290	-0.1343
Helotiaceae	0.2347	0.1327	0.7048	0.0425	0.4014	-0.0939	0.0390	-0.2285	0.0134	-0.2721	0.0192	-0.2581	0.0576	-0.2106	0.0240	-0.2492
Fomitopsidaceae	0.6593	0.0494	0.7778	0.0316	0.9148	-0.0120	0.6280	0.0543	0.1614	0.1561	0.8736	-0.0178	0.2659	-0.1243	0.9343	0.0093
Diatrypaceae	0.2959	-0.1260	0.1624	0.1557	0.2740	0.1222	0.8502	0.0212	**0.0231**	**0.2508**	0.1672	0.1540	0.1009	0.1825	0.7172	0.0406
Polyporaceae	0.3879	0.0966	0.2543	0.1273	0.7065	0.0422	0.5447	-0.0678	0.9699	0.0042	0.7218	-0.0399	0.5242	-0.0713	0.0918	-0.1873
Togniniaceae	0.1119	-0.1769	0.0079	-0.2915	0.1601	-0.1666	0.5896	-0.0604	0.3049	-0.1147	0.8477	-0.0215	0.3798	0.0983	0.8943	-0.0149
Ganodermataceae	0.6106	0.0571	0.9777	-0.0032	0.1159	0.1750	0.6947	0.0440	0.5040	-0.0748	0.1301	0.1686	0.6432	0.0519	0.6678	0.0481
Mycenaceae	0.0273	-0.2437	0.1315	-0.1680	0.0014	-0.3466	0.0103	-0.2820	0.0468	-0.2203	0.1052	-0.1802	0.0020	-0.3370	0.5659	-0.0643
Helotiales_I.S.	0.1713	0.1525	0.1335	-0.1671	0.6181	-0.0559	0.1769	-0.1506	0.2092	-0.1402	0.0608	-0.2080	0.0845	-0.1917	0.9623	-0.0053
Schizoporaceae	0.8464	0.0217	0.2281	0.1346	0.2650	0.1245	0.3763	-0.0990	0.4041	-0.0934	0.2981	-0.1163	0.3601	-0.1024	0.0011	-0.3532
Stereaceae	0.2670	0.1240	0.7087	0.0419	0.2442	-0.1301	0.2203	-0.1368	0.7020	-0.0429	0.4997	-0.0756	0.0538	-0.2138	0.0036	-0.3179
Chaetosphaeriaceae	0.5954	-0.0595	0.0922	-0.1872	0.5102	-0.0738	0.0723	-0.1996	0.0621	-0.2070	0.2620	-0.1253	0.0820	-0.1932	0.4159	-0.0911
Psathyrellaceae	0.4188	0.0905	0.1274	0.1697	0.8740	0.0178	0.4257	0.0892	0.7785	-0.0315	0.9098	0.0127	0.5968	-0.0593	0.4047	-0.0932
Bondarzewiaceae	0.0385	-0.2291	0.6430	-0.0520	0.4833	-0.0785	0.6958	0.0438	0.1129	-0.1764	0.5339	-0.0697	0.8868	0.0160	0.8201	0.0255
Exidiaceae	0.7501	0.0357	0.4926	-0.0768	0.7792	-0.0314	0.0287	-0.2417	0.0093	-0.2855	0.1263	-0.1702	0.2291	-0.1342	0.0082	-0.2900
Amylostereaceae	0.4270	-0.0890	0.2243	0.1356	0.6293	-0.0541	0.0294	-0.2497	0.0824	-0.1930	0.0934	-0.1865	0.0912	-0.1877	0.0262	-0.2455
Tympanidaceae	0.5154	-0.0729	0.0036	-0.3180	0.0095	-0.2846	0.0141	-0.2701	0.0065	-0.2983	0.0025	-0.3289	0.0487	-0.2184	0.3770	-0.0988
**eco-types**																
WRF	0.4141	-0.0914	**0.0165**	**0.2640**	**0.0073**	**0.2942**	0.7482	0.0359	0.2631	-0.1250	0.3800	-0.0982	0.1542	-0.1283	0.5416	-0.0684
BRF	0.1401	-0.1644	0.2926	-0.1176	0.4005	-0.0941	0.8275	0.0244	0.5608	0.0652	0.3482	-0.1049	0.3955	-0.0951	0.6291	0.0541
SRF	0.4015	0.0939	0.9217	-0.0110	0.1881	-0.1468	0.9723	0.0039	0.9952	-0.0007	0.3356	0.1077	0.5353	0.0694	0.9187	0.0114

most abundant families	ß-N-acetyl-hexosaminidase		Leucine-aminopeptidase		ß-D-glucuronidase		α-D-mannosidase		α-L-arabinosidase		Acid phosphatase		Sulfatase	
	p	ϱ	p	ϱ	p	ϱ	p	ϱ	p	ϱ	p	ϱ	p	ϱ
Meruliaceae	0.3827	0.0977	0.1125	0.1766	0.6681	0.0481	0.3011	-0.1156	0.1151	0.1754	0.5086	0.0740	0.5902	-0.0603
Coniochaetaceae	**0.0037**	**0.3170**	0.9848	0.0021	0.1934	0.1451	**0.0194**	**0.2577**	0.0356	-0.2322	**0.0034**	**0.3202**	0.1785	0.1500
Herpotrichiellaceae	0.9803	0.0028	0.0493	-0.2179	0.5654	-0.0644	0.9606	0.0055	0.9963	-0.0005	0.4455	0.0854	0.6075	-0.0576
Xylariaceae	0.1930	-0.1452	0.3955	-0.0951	0.1394	-0.1646	0.1561	-0.1581	0.2275	0.1347	0.4836	-0.0784	0.3396	-0.1068
Hypoxylaceae	0.0778	0.1959	0.6288	0.0542	0.1320	0.1678	0.8181	0.0258	0.1019	0.1819	**0.0382**	**0.2294**	0.5486	0.0672
Strophariaceae	0.0957	-0.1853	0.0078	-0.2921	0.0940	-0.1862	0.4118	-0.0919	0.3918	-0.0958	$4.827\ e^{-05}$	-0.4331	0.4612	-0.0825
Helotiaceae	0.0226	-0.2516	0.1896	-0.1463	0.0307	-0.2388	0.2419	-0.1307	0.4259	-0.0891	0.3951	-0.0951	0.0450	-0.2220
Fomitopsidaceae	0.8039	-0.0278	0.8264	0.0246	0.1963	0.1442	0.1557	0.1582	0.4959	-0.0763	0.0854	0.1911	0.1286	0.1692
Diatrypaceae	0.5255	0.0711	0.3497	-0.1046	0.8469	-0.0217	0.1673	-0.1540	0.3502	0.1045	0.1823	0.1488	0.5223	-0.0717
Polyporaceae	0.3086	-0.1138	0.8526	0.0208	0.6412	0.0522	0.5129	0.0732	0.8253	-0.0247	0.1207	0.1727	0.7387	-0.0374
Togniniaceae	0.2348	0.1327	0.2160	-0.1381	0.6370	-0.0529	0.3618	0.1020	0.0575	-0.2107	0.6807	-0.0461	0.6598	0.0493
Ganodermataceae	0.5519	0.0666	0.5206	0.0720	0.6708	-0.0476	0.5945	0.0596	0.8819	-0.0166	0.4719	-0.0805	0.8924	0.0152
Mycenaceae	0.0480	-0.2191	0.4677	-0.0813	0.3361	-0.1076	0.6617	-0.0490	0.4741	-0.0802	0.4475	-0.0850	0.7720	0.0325
Helotiales_I.S.	0.7813	-0.0311	0.2370	0.1095	0.7705	-0.0327	0.6013	0.0586	0.0720	-0.1997	0.8399	0.0227	0.7274	-0.0371
Schizoporaceae	0.2244	-0.1356	0.0827	-0.1928	0.4549	-0.0837	0.3222	-0.1107	**0.0203**	**0.2559**	0.4729	-0.0804	0.0694	-0.2015
Stereaceae	0.1010	-0.1824	0.0218	-0.2531	0.0867	-0.1904	0.6292	0.0541	0.9321	-0.0096	0.6319	0.0537	0.5080	-0.0741
Chaetosphaeriaceae	0.6489	-0.0510	0.8711	0.0182	0.8137	0.0264	0.4936	0.0767	0.0836	-0.1922	0.4988	-0.0757	0.5845	0.0613
Psathyrellaceae	0.8104	-0.0269	0.8824	-0.0166	0.6100	-0.0572	0.0835	-0.1923	**0.0081**	**0.2904**	0.5638	-0.0647	0.2363	-0.1323
Bondarzewiaceae	0.7985	-0.0286	0.2923	-0.1177	0.9301	0.0098	0.9317	-0.0096	0.8561	-0.0203	0.0325	-0.2364	0.5921	-0.0600
Exidiaceae	0.1868	-0.1473	0.0518	-0.2156	0.0051	-0.3068	0.1752	-0.1512	0.9736	-0.0037	0.0072	-0.2948	0.0201	-0.2563
Amylostereaceae	0.0290	-0.2413	0.6418	-0.0521	0.0878	-0.1807	0.0543	-0.2133	0.6093	0.0573	0.0094	-0.2851	0.0475	-0.2195
Tympanidaceae	0.0689	-0.2019	0.4223	-0.0898	0.3321	-0.1085	0.7831	-0.0309	0.1175	-0.1742	0.6159	-0.0562	0.5017	-0.0752
**eco-type**														
WRF	0.2504	-0.1283	0.3282	0.1093	0.6475	-0.0512	0.8016	-0.0282	0.8591	-0.0199	0.0640	-0.2055	0.2566	-0.1267
BRF	0.5363	-0.0693	0.6859	0.0453	0.8690	0.0184	0.8676	0.0187	0.6738	0.0472	0.5400	0.0686	0.3148	0.1124
SRF	0.7204	-0.0401	0.1861	-0.1475	0.4859	-0.0780	0.7903	-0.0298	0.4389	-0.0867	0.1310	0.1682	0.6557	-0.0499
